# Comparison of inflammatory molecular mechanisms between osteoarthritis and rheumatoid arthritis via gene microarrays

**DOI:** 10.22099/mbrc.2024.49924.1963

**Published:** 2024

**Authors:** Maziar Oveisee, Akram Gholipour, Mahshid Malakootian

**Affiliations:** 1 Orthopedic Department, Bam University of Medical Sciences, Bam, Iran; 2 Cardiogenetic Research Center, Rajaie Cardiovascular Medical and Research Center, Iran University of Medical Sciences, Tehran, Iran; # These authors have equally contributed to this work.

**Keywords:** Rheumatoid arthritis. Osteoarthritis. Differentially expressed genes. Functional analysis. Inflammatory signaling pathway

## Abstract

Osteoarthritis (OA) and rheumatoid arthritis (RA) treatment requires exact arthritis type diagnosis. We compared inflammatory molecular mechanisms between OA and RA to introduce reliable molecular biomarkers. The GSE55235 and GSE100786 microarray datasets were acquired from the GEO. Data preprocessing and differential expression analysis were conducted in OA and RA groups and their control groups applying GEO2R. Differentially expressed genes (DEGs) with a |LogFC|>1 and adj. *p*<0.05 were determined. Gene ontology (GO) and signaling pathway analysis were done utilizing PANTHER and Enrichr. The suitability of gene expression alterations as biomarkers was tested using the receiver operating characteristic (ROC) curve analysis. We found 2129 DEGs between the OA and control groups and 2494 DEGs between the RA and control groups. GO on the DEGs showed enrichment in binding, cellular processes, and cellular anatomical entities in molecular functions, biological processes, and cellular components, respectively. Enrichr found the cell differentiation pathways of Th1 and Th2 only in RA. The ROC curve analysis indicated *HLA-DQA1* downregulation and *MAPK8IP3 *upregulation as reliable biomarkers to discriminate RA from OA in peripheral blood and bone marrow samples, respectively. We found more DEGs in patients with OA than those with RA and determined inflammatory pathways and genes unique to RA as reliable biomarkers to discriminate RA from OA. Gene expression alterations associated with Th1 and Th2 cell differentiation pathways, including *HLA-DQA1 *downregulation and *MAPK8IP3* upregulation, could be novel molecular biomarkers to diagnose RA.

## INTRODUCTION

Arthritis is a severe and persistent disease encompassing a variety of disorders, with joint pain as a common characteristic. The 2 most common forms of arthritis with different underlying mechanisms are osteoarthritis (OA) and rheumatoid arthritis (RA) [1]. OA is a degenerative disease of the joints among aging individuals that predominantly affects the hands, hips, and knees. OA happens when the cartilage or the cushion between the joints breaks and causes pain, swelling, and stiffness [2]. It also manifests itself with articular cartilage loss, synovial membrane inflammation, cartilage surface fibrillation, abnormal articular chondrocyte differentiation, bone remodeling, and matrix proteoglycan depletion [3-5].

RA is a common systemic autoimmune disease. A progressive, chronically inflammatory, injurious joint disease primarily affecting the small joints of the hand and feet, RA occurs when the immune system invades the joints, generates inflammation, and begets joint thickness [6]. It is also characterized by chronic synovial inflammation, cartilage demolition, and bone destruction, resulting in permanent disability due to swelling and pain in and around the joints [6, 7].

Although OA and RA share such features as joint dysfunction and soreness and possess overlapping cellular and molecular mechanisms, they have different pathogeneses. In a routine clinical evaluation, semiquantitative approaches, including radiological imaging, synovitis histopathological assessment, symptom determination, physical function, laboratory values such as rheumatoid factors and citrullinated peptides, rheumatic nodule detection, and joint damage assessment, as well as family history taking, are employed to diagnose RA and OA [3, 8-12]. 

On the one hand, profiling RNA expression in 2 different samples can illustrate how genes are expressed in physiological and pathophysiological states [13-15], and on the other hand, identifying differentially expressed genes (DEGs) between disease and healthy states will shed further light on gene signatures, biomarkers, and therapeutic targets [13, 16-21].

The difference in the approaches to OA and RA treatment renders the accurate diagnosis of the arthritis type significant. Indubitably, utilizing a noninvasive modality to distinguish biomarkers between these 2 joint diseases is scintillating. Accordingly, in the present study, we drew upon bioinformatics to determine the commonly and differentially expressed genes in OA and RA.

## MATERIALS AND METHODS

**Datasets: **The National Center for Biotechnology Information’s Gene Expression Omnibus (GEO) database was employed to download appropriate microarray datasets containing GSE55235 and GSE100786 accession numbers. The samples obtained from the GSE55235 microarray analysis comprised 10 normal samples, 10OA samples, and 10RA samples of synovial tissue from joint. The samples acquired from the GSE100786 microarray analysis were composed of 8 samples of monocytes from the bone marrow samples of RA patients, 8 samples of monocytes from the bone marrow samples of OA patients, 6 samples of monocytes from the peripheral blood samples of RA patients, and 6 samples of monocytes from the peripheral blood samples of OA patients. The GSE100786 dataset samples were used to confirm the expression of the inflammatory genes indicated as candidates by the GSE55235 dataset analysis.

**Differential gene expression analysis: **The expression profiles of the genes differentially expressed between an OA group and a control group and an RA and a control group were analyzed by GEO2R (https://www.ncbi.nlm.nih.gov/geo/geo2r). Statistical parameters to determine genes with significant expression differences in the datasets were a |LogFC|>1 and a adj. *p*<0.05. 

**Gene ontology (GO) enrichment analysis of the DEGs:** Annotations of the cellular components, biological processes, and molecular functions of DEGs with a |LogFC|>2 in each group were analyzed using the PANTHER (http://www.pantherdb.org/) database. 

**Functional analysis of genes with the most expression changes: **For the identification of significant inflammatory signaling pathways with the most changes in gene expression and the ability to differentiate between OA and RA, genes with a |LogFC|>2 in each of the OA and RA groups were isolated, and significant (adj. *p*<0.001; adjusted *p* value used when one performs multiple comparisons in a more general sense) inflammatory signaling pathways were checked using the Enrichr database (https://maayanlab.cloud/Enrichr/enrich) in the KEGG section. 

**Inflammatory pathway evaluation and gene expression confirmation in OA and RA: **Further analysis was conducted by selecting the most statistically significant inflammatory pathways (adj. *p*<0.001) that were not common between the OA and RA groups. Gene expression in the selected pathway was examined in different samples of the GSE100786 dataset. DEGs in this dataset were also analyzed using GEO2R (https://www.ncbi.nlm.nih.gov/ geo/geo2r).

**Receiver operating characteristic (ROC) curve analysis: **A ROC curve analysis was conducted to determine whether the DEGs in the GSE100786 dataset had enough sensitivity and specificity to discriminate RA from OA. 

**Statistical Analysis: **The DEGs in the datasets were analyzed using the GEO2R database. The significance of differences in the expression levels of the selected genes in the synovial fluid between the RA and OA groups was determined using the GraphPad prism v.8.1 software. A adj. *p*-value <0.05 was considered statistically significant. In the pathway functional and GO enrichment analyses, an adj. *p*-value<0.001 was considered statistically significant.

## RESULTS

**Figure 1:**
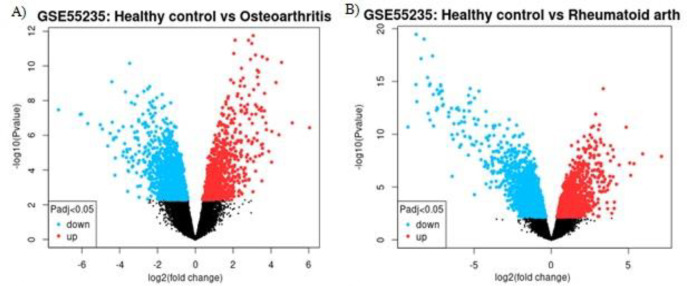
The image presents A) genes whose expression was significantly different between the OA group and a control group B) and between the RA group and a control group. Genes with significant differential expression were considered via the |LogFC|>1 and adj.*p*<0.05 parameters. OA, Osteoarthritis; RA, Rheumatoid arthritis.

The analysis of the GSE55235 dataset showed that 2129 genes and 2494 genes were significantly differentially expressed between the OA and control groups and between the RA and control groups, respectively. The volcano plot is illustrated in Figure 1A-B, and the names are listed in Supplementary Tables S1 and S2. The GO enrichment analysis demonstrated that DEGs were principally enriched in cellular components, biological processes, and molecular functions in the RA and control groups and the OA and control groups.

Additionally, DEGs from the RA and OA groups were commonly enriched in GO terms, including molecular functions associated with binding, catalytic activities, molecular transducer activities, molecular function regulations, and transcription regulator activities. In addition, DEGs were associated with biological processes, including cellular processes, biological regulations, metabolic processes, responses to stimuli, and signaling. Moreover, DEGs were enriched vis-à-vis cellular components, including cellular anatomical entities and protein-containing complexes in both RA and OA groups. Tables 1 and 2 presents all GO analysis terms and their related genes. 

**Table 1:** Normal - OA important GOs enrichment with top 10 genes in each description

**GO term description**	**ID**	**Genes**
**Molecular Function (MF): ** binding catalytic activity molecular transducer activity molecular function regulator transcription regulator activity	GO:0005488 GO:0003824 GO:0060089 GO:0098772 GO:0140110	*LBP, CDH2, FOSL1, RHOB, KLF9, NR4A3, HTR2B, TIMP4, PSPH, AIF1,…* *LBP, RHOBM, TIMP4, PPP1R15A, DUSP2, PTGS2, SIK1, PIGA, MMP9, FADS1,…* *HTR2B, TNFRSF11A, CXCL3, TLR7, IL1B, LILRB2, GPR65, RAMP2, PLXNC1, PTN,…* *TIMP4, PPP1R15A, CXCL3, IL1B, PTN, CCL5, CCL20, WNT5B, VEGFA, CCNL1,…* *FOSL1, FOSL1, NR4A3, ZBTB43, FOXO3, FOSB, THRA, KLF4, NR4A1, IKZF1,…*
**Biological Process (BP): ** cellular process biological regulation metabolic process response to stimulus signaling	GO:0009987 GO:0065007 GO:0008152 GO:0050896 GO:0023052	*LBP, CDH2, TTC3, FOSL1, KLF9, NR4A3, HTR2B, TIMP4, PSPH, TOP2A,…* *LBP, CDH2, FOSL1, KLF9, NR4A3, HTR2B, TIMP4, CKAP2, IGHD, TNFRSF11A,…* *TTC3, FOSL1, KLF9, NR4A3, TIMP4, PSPH, IGKV1OR2-108, TOP2A, IGHD, TNFRSF11A,… * *LBP, NR4A3, HTR2B, TIMP4, IGKV1OR2-108, IGHD, CX3CR1, TNFRSF11A, PPP1R15A, CXCL3,…* *LBP, CDH2, HTR2B, IGHD, TNFRSF11A, CXCL3, ADM, TLR7, IL1B, DUSP2,…*
**Cellular Component (CC):** cellular anatomical entity protein-containing complex	GO:0110165 GO:0032991	*LBP, CDH2, FOSL1, NR4A3, HTR2B, TIMP4, PSPH, IGKV1OR2-108, TOP2A, AIF1,…* *CDH2, NR4A3, IGHD, KCNQ1, PPP1R15A, HLA-DQB1, RPS4Y1, HLA-DMB, RAMP2, PIGA,…*

DEGs with a |LogFC|> 2 and a adj. *p*<0.05 were examined using the KEGG database. The results revealed that the interleukin-17 (IL-17) signaling, nuclear factor kappa B (NF-κB), cytokine-cytokine receptor interaction, and T helper 17 (Th17) cell differentiation pathways were the common pathways between OA and RA. Table 3 lists the common significant inflammatory pathways between the 2 diseases.

Moreover, the Th1 cell differentiation, Th2 cell differentiation, B cell receptor signaling, primary immunodeficiency, and T cell receptor signaling pathways were only involved in RA. Thus, these pathways and their involved genes discriminated RA from OA at the molecular level. The Th1 and Th2 cell differentiation pathways were the most statistically significant of these pathways (adjusted *p*=1.227e-10) in RA. Consequently, the deregulated expression of genes (Table 4) in this pathway might play a considerable role in RA pathogenesis. The expression of the genes involved in the Th1 and Th2 cell differentiation pathways was also examined in the blood datasets of OA and RA patients (Table 4). 

**Table 2:** Normal-RA important GOs enrichment with top 10 genes in each description

**GO term description**	**ID**	**Genes**
**Molecular Function (MF): ** binding catalytic activity molecular transducer activity molecular function regulator transcription regulator activity	GO:0005488 GO:0003824 GO:0060089 GO:0098772 GO:0140110	*HLA-DOB, FOSL1, CST7, ITGAX, KLF9, ERAP2, RTP4, TIMP4, AIF1, IRS2,…* *CST7, ERAP2, TIMP4, JAK2, APOBEC3F, PDK4, SYK, ATP2A3, TRHDE, PPP1R15A,…* *SEMA4D, CXCL13, IL7R, TLR7, NLGN1, NPY1R, LILRB2, CX3CL1, GPR65, RAMP2, …* *CST7, TIMP4, SEMA4D, CXCL13, PPP1R15A, CX3CL1, FIGF, CCL5, CXCL9,…* *FOSL1, KLF9, ZEB1, BCL11A, FOXO3, PAX5, FOSB, THRA, KLF4, TRAC,…*
**Biological Process (BP): ** cellular process biological regulation metabolic process response to stimulus signaling	GO:0009987 GO:0065007 GO:0008152 GO:0050896 GO:0023052	*HLA-DOB, KLRD1, FOSL1, CST7, ITGAX, KLF9, ERAP2, RTP4, TIMP4, SCN9A,…* *HLA-DOB, KLRD1, FOSL1, CST7, ITGAX, KLF9, CR1, TIMP4, SCN9A, CD79A,…* *HLA-DOB, FOSL1, CST7, KLF9, CR1, ERAP2, TIMP4, IGKV1OR2-108, TOP2A, IGHD,…* *HLA-DOB, KLRD1, CST7, ITGAX, CR1, TIMP4, IGKV1OR2-108, CD79A, IRS2, IGHD,…* *KLRD1, ITGAX, CD79A, IRS2, IGHD, JAK2, SEMA4D, ITGB7, OLFM4, CXCL13,…*
**Cellular Component (CC):** cellular anatomical entity protein-containing complex	GO:0110165 GO:0032991	*HLA-DOB, FOSL1, CST7, ITGAX, CR1, ERAP2, TIMP4, SCN9A, IGKV1OR2-108, CD79A,…* *HLA-DOB, ITGAX, SCN9A, CD79A, IGHD, HLA-DMA, ITGB7, KCNQ1, PPP1R15A, HLA-DQB1*

**Table 3:** Important inflammatory pathways, Number 1-9: Inflammatory pathways among OA-Normal, Number 10-22: Inflammatory pathways among RA-Normal. Th1 and Th2 cell differentiation pathway is a unique pathway in RA

**Number**	**Name**	**Adjusted ** ***p*** **-value**
1	IL-17 signaling pathway	2.138e-10
2	NF-kappa B signaling pathway	6.498e-10
3	TNF signaling pathway	1.292e-7
4	Cytokine-cytokine receptor interaction	0.00001296
5	Chemokine signaling pathway	0.0001352
6	Intestinal immune network for IgA production	0.0003740
7	Toll-like receptor signaling pathway	0.0005890
8	Th17 cell differentiation	0.003148
9	Antigen processing and presentation	0.003244
10	Cytokine-cytokine receptor interaction	1.973e-11
11	Th17 cell differentiation	1.973e-11
12	Th1 and Th2 cell differentiation	1.227e-10
13	Intestinal immune network for IgA production	2.118e-10
14	Antigen processing and presentation	1.033e-9
15	IL-17 signaling pathway	1.059e-8
16	Chemokine signaling pathway	3.818e-8
17	TNF signaling pathway	6.936e-7
18	B cell receptor signaling pathway	0.000006396
19	NF-kappa B signaling pathway	0.00006698
20	Toll-like receptor signaling pathway	0.00006698
21	Primary immunodeficiency	0.000006524
22	T cell receptor signaling pathway	0.005372

**Table 4:** Th1 and Th2 cell differentiation pathway genes in RA disease

**Number**	**Genes**	**LogFC**	**p.adj.Value**
1	*JUN*	3/33828555	2/55E-05
2	*HLA-DRB4*	-3/1704995	1/28E-02
3	*STAT1*	-2/96193376	6/49E-08
4	*FOS*	2/22374506	1/88E-04
5	*CD3D*	-2/56324388	3/49E-09
6	*HLA-DMA*	-2/19770466	8/07E-09
7	*MAPK8*	2/024939	7/62E-05
8	*HLA-DMB*	-2/73003	4/10E-12
9	*LCK*	-3/28592631	2/48E-07
10	*HLA-DPB1*	-2/1002	4/35E-09
11	*HLA-DRA*	-2/33298	1/99E-07
12	*CD247*	-2/35075	1/34E-10
13	*JAK2*	-2/18762	1/10E-04
14	*HLA-DOB*	-3/64263	1/53E-11
15	*HLA-DQA1*	-3/54835	1/14E-02
16	*HLA-DPA1*	-2/8317	5/05E-09

**Figure 2:**
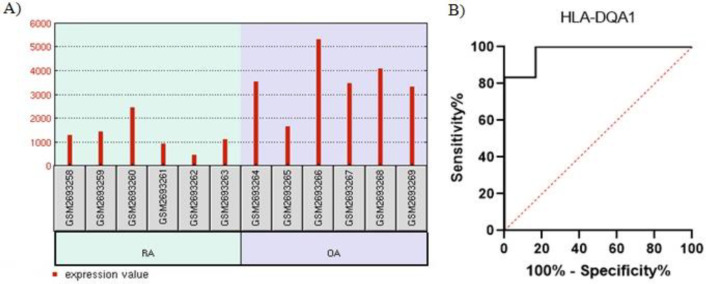
The image illustrates A) *HLA-DQA1* downregulation in the peripheral blood samples of the group with rheumatoid arthritis and B) the receiver operating characteristic (ROC) curve analysis of *HLA-DQA1.*

The *HLA-DQA1* showed significant downregulation in the peripheral blood samples of RA patients (logFC = −1.542, adjusted *p*=0.0015 (Fig. 2A). Furthermore, the results of the ROC curve analysis confirmed the reliability of this marker in differentiating RA from OA (area under the curve [AUC], 0.97; 95% CI, 0.88-1; *p*=0.006) with a sensitivity of 100% and a specificity of 83.33% (cutoff >1559; the absolute value of the difference between the sensitivity and specificity) (Fig. 2B). 

**Figure 3:**
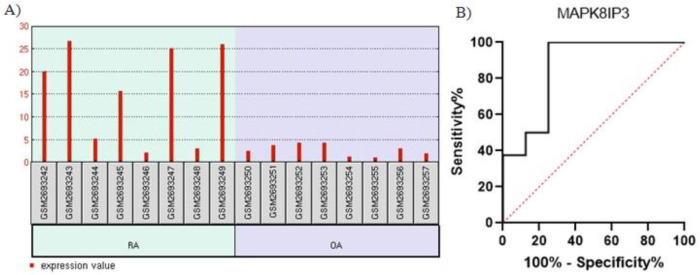
The image shows A) *MAPK8IP3* upregulation in the bone marrow samples of the group with rheumatoid arthritis and B) the receiver operating characteristic (ROC) curve analysis of *MAPK8IP3.*

The gene expression analysis of the Th1 and Th2 cell differentiation pathways in the bone marrow samples of RA and OA patients in the GSE100786 dataset revealed that *MAPK8IP3*, a component of the MAPK8 pathway, had significant upregulation in the RA group (logFC =2.066, adjusted *p*=0.0019 (Fig. 3A). Further, the ROC curve analysis confirmed the reliability of *MAPK8IP3 *expression as a molecular marker in differentiating RA from OA in bone marrow samples (AUC, 0.85; 95% CI, 0.66-1; *p*=0.015) with a sensitivity of 100% and a specificity of 75% (cutoff <4.95) (Fig. 3B). The analysis of this dataset also confirmed that these inflammatory pathways were involved in the pathogenesis of both RA and OA and could, therefore, distinguish them at the molecular level.

## DISCUSSION

While OA and RA are engendered by different mechanisms, in the present study, we found inflammatory pathways common between the 2 arthritis forms. Nevertheless, our results also demonstrated inflammatory pathways that were only present in RA. 

We performed the current investigation based on the hypothesis that both inflammatory pathways and the differential expression of the genes involved could discriminate RA from OA at the molecular level. Based on our findings, *HLA-DQA1* downregulation and *MAPK8IP3 *upregulation in the monocytes of peripheral blood and bone marrow samples can distinguish RA from OA. Moreover, our ROC curve analysis results indicated that the differential expression of *HLA-DQA1* and *MAPK8IP3* could be potential biomarkers of both arthritis forms. 

Gene expression profiling analysis is now extensively utilized to improve diagnosis and find novel pathways involved in the pathogenesis of different diseases, such as cancers [22, 23], intervertebral  disc degeneration [20], carpal tunnel syndrome, OA, and RA [24-27]. Previous investigations have demonstrated some DEGs in RA and OA samples compared with normal ones [28-31]. 

We detected more DEGs in the OA group than the RA group, which is consistent with the findings of Zuo et al [32, 33] and Del Rey et al [31]. Additionally, our GO enrichment analysis revealed that signaling was an integral component of biological processes in both RA and OA: 8 DEGs (*KLRD1, ITGAX, CD79A, IRS2, JAK2, SEMA4D, ITGB7*, and *OLFM4*) screened from the RA group and 8 DEGs (*LBP, CDH2, HTR2B, TNFRSF11A, ADM, TLR7, IL1B*, and *DUSP2*) screened from the OA group played significant roles in signaling biological processes. 

Lu et al [34] showed that KLRB1 could be key mediators of RA pathogenesis and markers of RA diagnosis. Furthermore, *ITGAX* is considered a risk gene in autoimmune diseases [35], and synovial CD79a-positive B cells may be a helpful biomarker of histologic disease activities in RA [36, 37]. Yoshida et al [38] posited that a positive feedback loop comprising sSema4D/IL-6 and TNFα/ADAMTS-4 might contribute to RA pathogenesis, and Ren et al [39] concluded that *OLFM4* played a significant role in joint inflammation in RA. 

Huang et al [40] showed that plasma *LBP* and *sTLR4* were associated with OA progression in the knee. In this regard, *CDH2* polymorphisms are risk factors for knee OA [41, 42]. Mutations of *JAK2* may be involved in the response and help treatment of RA [43]. The *HTR2B* gene is a specific marker in age-related OA via the apoptosis and inflammation of OA synovial cells [44]. Hu et al [45] showed that *TLR7* was associated with various immune cells and was a potential diagnostic marker and therapeutic target for OA. Research has also shown the hugely influential role of IL-1 in the discovery of proteases responsible for cartilage degradation in OA [46, 47]. 

Our findings concerning the significance of inflammatory pathways common between OA and RA are in concordance with previous investigations. However, to our knowledge, we are the first to introduce specific genes as biomarkers in those pathways. 

In addition, we found that different inflammatory pathways were among critical pathways in both RA and OA, which is in line with the findings of Sun et al [48, 49]. Li et al [50] demonstrated that the chemokine signaling, cytokine‐cytokine receptor interaction, and cytosolic DNA-sensing inflammatory pathways underlay RA and OA pathogenesis. 

Both OA and RA are accompanied by joint inflammation [48, 51]. As was stated above, our results chime in with those previously reported insofar as some inflammatory pathways are involved in both RA and OA. Still, we succeeded in identifying the Th1 cell differentiation, Th2 cell differentiation, B cell receptor, primary immunodeficiency, and T cell receptor signaling pathways, which are involved only in RA.

Ge et al [52] underscored the significance of cytokine receptor interaction and immune infiltration in RA development, and Cavalli et al [53] highlighted the key role of IL-37 in suppressing joint and systemic inflammation in arthritis. Zhang et al [54] demonstrated an association between the B cell receptor signaling, toll-like receptor signaling, and Fc gamma R-mediated phagocytosis pathways and RA. In another study, Zhang et al [31] reported a likely association between RA occurrence and elevated expression levels of *IL7R* and *STAT1* in synovial tissue and primary immunodeficiency. 

Naive T cells can differentiate into different subsets based on the signals with which they are faced. The differentiation of Thl/Th2 cells exerts a drastic impact on the inflammatory response and plays a significant role in autoimmune diseases [55]. RA is thought to be a T cell-mediated disease. NKT cells not only have a paramount role in RA development by polarizing Th1, Th2, Th17, and Treg cells but also influence it by regulating Th cell differentiation [56, 57]. An imbalance between Th1/Th2 and Th17/Treg cells may be responsible for the occurrence and development of RA [58, 59]. 

 Our results revealed that the Th1 and Th2 cell differentiation pathways could constitute the most statistically significant pathways in RA (adjusted *P*=1.227e-10). Consequently, we suggest that the differential expression of *JUN, HLA-DRB4, STAT1, FOS, CD3D, HLA-DMA, MAPK8, HLA-DMB, LCK, HLA-DPB1, HLA-DRA, CD247, JAK2, HLA-DOB, HLA-DQA1, HLA-DPA1*, involved in these pathways, might be a suitable marker to distinguish RA from OA. On this point, a previous bioinformatics study demonstrated that *CD247* and *LCK* were differentially expressed in RA [52]. Via an *in silico *analysis, Zhang et al [31] posited that *STAT1* might be associated with RA, concordant with our results regarding the introduced genes. 

In the present study, we analyzed peripheral blood and bone marrow sample datasets of RA and OA patients and demonstrated the expression of the genes involved in the Th1 and Th2 cell differentiation pathways. According to our synovial tissue dataset analysis, *HLA-DQA1* expression was downregulated in the peripheral blood samples of RA patients, whereas *MAPK8IP3* expression was upregulated in the bone marrow samples of RA patients. Using human leukocyte antigen (HLA) typing, Guo et al [60] showed that HLA-DRB1*0405 was a strong and independent gene risk for RA in Han Chinese.

An optimal biomarker should have high sensitivity and specificity [61]. Our ROC curve and bioinformatics analysis data demonstrated that the aforementioned *HLA-DQA1* downregulation at the RNA level in peripheral blood samples and *MAPK8IP3* upregulation at the RNA level in bone marrow cells could be considered suitable and reliable noninvasive biomarkers for RA diagnosis. However, our results should be further investigated and confirmed by experimental analyses.
